# Poly-L-Lysine and Human Plasmatic Fibronectin Films as Proactive Coatings to Improve Implant Biointegration

**DOI:** 10.3389/fbioe.2021.807697

**Published:** 2022-01-17

**Authors:** Anamar Miranda, Damien Seyer, Carla Palomino-Durand, Houda Morakchi-Goudjil, Mathilde Massonie, Rémy Agniel, Hassan Rammal, Emmanuel Pauthe, Adeline Gand

**Affiliations:** ^1^ Equipe de Recherche sur les Relations Matrice Extracellulaire Cellules, Institut des Matériaux, CY Cergy-Paris Université, Cergy-Pontoise, France; ^2^ EFOR Healthcare Paris, Biocompatibility Platform, Levallois-Perret, France

**Keywords:** multilayer thin films, fibronectin, biomimetic, bioadhesive, antimicrobial activity

## Abstract

The success of stable and long-term implant integration implies the promotion, control, and respect of the cell microenvironment at the site of implantation. The key is to enhance the implant–host tissue cross talk by developing interfacial strategies that guarantee an optimal and stable seal of soft tissue onto the implant, while preventing potential early and late infection. Indeed, implant rejection is often jeopardized by lack of stable tissue surrounding the biomaterial combined with infections which reduce the lifespan and increase the failure rate of implants and morbidity and account for high medical costs. Thin films formed by the layer-by-layer (LbL) assembly of oppositely charged polyelectrolytes are particularly versatile and attractive for applications involving cell–material contact. With the combination of the extracellular matrix protein fibronectin (Fn, purified from human plasma) and poly-L-lysine (PLL, exhibiting specific chain lengths), we proposed proactive and biomimetic coatings able to guarantee enhanced cell attachment and exhibiting antimicrobial properties. Fn, able to create a biomimetic interface that could enhance cell attachment and promote extracellular cell matrix remodeling, is incorporated as the anionic polymer during film construction by the LbL technic whereas PLL is used as the cationic polymer for its capacity to confer remarkable antibacterial properties.

## 1 Introduction

The trend to replace damaged or diseased biological materials by artificial organs is a current challenge in regenerative medicine. More particularly, in the case of tooth loss or limb amputation, percutaneous and permucosal implants, like dental implants, and intraosseous transcutaneous amputation prosthesis (ITAP) can be used to replace the missing organ. The use of dental implants has been generalized in biomedical treatments. The implantation of both dental implants and ITAP is expected to drastically increase in the coming years (Elani et al., 2018; Hoellwarth et al., 2020). These implants have the particularity of being anchored into the bone tissue and to breach the soft tissue barrier that protects the body from the outer environment. Owing to its high resistance to corrosion, biocompatibility, non-cytotoxicity, and mechanical properties, titanium (Ti) and Ti alloys are materials of choice for many orthopedic/dental implants and fixation devices (Mantripragada et al., 2013; Liu et al., 2017). The main issue related to these implants is the poor soft tissue attachment leading to a breach that is open for bacterial colonization and thus infections compromising the long-term implantation of the device. For example, peri-implantitis incidence is estimated to occur in 20%–45% after the implantation of dental prosthesis (Dixon and London 2019). These complications can increase the failure rate of implants and account for high medical costs (Affeld et al., 2012).

To overcome this problem, different strategies were developed to avoid bacterial infection and biofilm formation. These strategies are mainly based on the use of polymers that present intrinsic antibacterial properties, like chitosan or polyethylene glycol, or serve as a reservoir for a controlled delivery of antimicrobial molecules and peptides, antibiotics, or silver (Michl et al., 2015; Ramiasa et al., 2015; Croes et al., 2018). Alternative strategies aim to enhance soft-tissue sealing around the implant and thus reduce bacterial invasions and related infection. This includes modifications of the topography—roughness, porosity, and nanostructuration (grooves, nanotubes)—that increases the surface area and thus the surface available for soft tissue attachment (Zitzmann et al., 2002; Puckett et al., 2010; Atsuta et al., 2014); modification of surface chemistry increasing surface hydrophilicity (Kloss et al., 2011; Abdallah et al., 2017); and grafting of biomolecules like proteins fibronectin (Fn) or laminin (Oyane et al., 2011; Ghadhab et al., 2021) and adhesion peptides like RGD or laminin-derived peptides (Bellis 2011; Fischer et al., 2020).

In the context of biomedical applications, coatings formed by layer-by-layer (LbL) assembly are of growing interest due to their high versatility (Gribova et al., 2012). This technic is based on the alternate deposition of oppositely charged polyelectrolytes (Decher et al., 1992) that results in the formation of thin coatings that are easy to fabricate and can be deposited on any type of surface irrespective of their geometry and chemistry which makes them attractive for implant functionalization. Thin film properties can be easily modulated in terms of thickness, rigidity, and roughness and can be functionalized with bioactive molecules or serve as a reservoir for the controlled delivery of biomolecules to confer antibacterial properties or to improve eukaryotic cell adhesion, proliferation, and differentiation (Guan et al., 2016; Hartmann and Krastev 2017; Shi et al., 2017; Ouni et al., 2021).

Here we propose a bifunctional biomimetic LbL coating, able to enhance soft tissue sealing and to prevent bacterial adhesion and subsequent biofilm formation, obtained by the association of Fn, and poly-L-lysine (PLL). Fn, a 220-kDa glycoprotein constitutive of the extracellular matrix (ECM), known to play a key role in cell adhesion, spreading, and proliferation, is used as the polyanion for its proadhesive properties (García et al., 1999; Webb et al., 2000; Hynes 2002) and PLL, known for its antibacterial properties (Conte et al., 2007; Dubois et al., 2013), as the polycation. In a previous study, using a high molecular weight PLL (70–150 kDa), we have shown that it is possible to build enriched Fn thin films (Gand et al., 2017). We demonstrated that these films presented a novel sub-linear terminated growth and could enhance murine MC3T3-E1 pre-osteoblastic cell adhesion, spreading, and proliferation compared to an Fn monolayer. Nevertheless, these thin films show no antibacterial properties, most probably due to the high molecular weight of the PLL that prevents the polyelectrolyte to diffuse within or out of the film.

In the present study, thin films were thus constructed using a lower molecular weight PLL of 30 residues (PLL_30_) and analyzed and challenged toward cell response: effect on primary gingival fibroblast behavior and antibacterial effects on three different strains: *Staphylococcus aureus*, *Staphylococcus epidermidis*, and *Streptococcus mutans*. These strains were specifically selected, as *S. aureus* and *S. epidermidis* are the most common pathogens encountered in implant-related infection, representing 2/3 of the infections (Campoccia et al., 2006), and the oral pathogen *S. mutans*, a gram-positive coccus, is considered as the major pathogen in early-stage biofilm formation and initiation of dental caries. Recent studies showed that *S. mutans* colonize the subgingival region with an increase in the case of chronic periodontal disease (Dani et al., 2016; Laosuwan et al., 2018).

## 2 Materials and Methods

### 2.1 Fn Preparation

Fn is purified from human plasma following a three-step affinity chromatography process, as previously described by Poulouin et al. (1999). This purification process has been shown to provide a protein solution exhibiting a purity of about 98% (w/w). The Fn concentration is determined by optical absorbance measurement A^1%^_280 nm_ = 12.8.

### 2.2 Thin Film Assembly

Films are constructed with Fn as polyanion and PLL with a specific chain length of 30 residues (2–3 kDa) as polycation either on 14-mm-diameter glass slides or on silicon crystals, pretreated with piranha solution (20% H_2_O_2_, 20% H_2_SO_4_). Briefly, film assembly occurs through an alternate deposition of 0.5 g/l PLL (PLL_30_, Alamanda Polymers, Huntsville, AL, USA) and 0.5 g/l Fn, in 5 mM phosphate buffer, pH 7.4. The exposure time was 5 min for PLL_30_ and 15 min for the first three layers of Fn and 8 min for the remaining layers. Each step is followed by three 1-min buffer rinse steps. For attenuated total reflection Fourier transform infrared spectroscopy (ATR-FTIR) experiments, PLL_30_ and Fn are dissolved in 5 mM deuterated phosphate buffer of pH 7.4, at a concentration of 0.5 g/l. For stimulated emission depletion (STED) microscopy experiments, samples were built on piranha-treated 12-mm (#1.5 thickness) glass coverslips, following the same procedure as previously mentioned.

### 2.3 Attenuated Total Reflection-Fourier Transform Infrared Spectroscopy

ATR-FTIR spectra were recorded using a Tensor 27 spectrophotometer (Bruker, Mannheim, Germany) equipped with an MCT detector. Polyelectrolyte and protein adsorption experiments were performed using a Horizontal ATR accessory (HATR, PIKE Technologies, Fitchburg, WI, USA) fitted with a 45° trapezoidal Silicon IRE (internal reflection element) crystal (80 mm × 10 mm × 2 mm). Protein and polyelectrolyte solutions in deuterated phosphate buffer were exposed to the crystal from 5 to 15 min, followed by 3 rinsing steps. All infrared spectra are an average of 64 collected scans recorded between 800 and 4,000 cm^−1^ with 2 cm^−1^ resolution, using Blackman–Harris three-term apodization and the Bruker OPUS software (version 7.2).

### 2.4 Scanning Electron Microscopy

Samples were washed with PBS (phosphate-buffered saline, Dulbecco’s^®^) 0.1 M pH 7.4 and fixed with glutaraldehyde 2% in PBS for 1 h at room temperature. After washing in 0.1 M cacodylate buffer pH 7.5, samples were dehydrated through a graded concentration of ethanol and critical point dried (CPD300, Leica, Wetzlar, Germany). Samples were mounted on SEM stubs, sputtered with 4 nm of Platinum (ACE600, Leica), and imaged using a field-emission gun scanning electron microscope (GeminiSEM 300, Carl Zeiss, Thornwood, NY, USA) with an acceleration voltage of 2 keV. Secondary electrons were collected.

### 2.5 Atomic Force Microscopy

Atomic force microscopy (BioScope Resolve, Bruker) was used to image the thickness, topography, and elasticity of the films in PBS 0.1 M pH 7.4 with a conical probe (70 nm radius, 0.112 N/m spring constant). Young’s Modulus values were recorded using the PeakForce Tapping mode at a rate of 2 kHz. Images were analyzed with Nanoscope Analysis software (Bruker).

### 2.6 Total Protein Quantification

Total protein quantification in (PLL-Fn)_10_ films was performed using bicinchoninic acid (BCA) assay as already described by Gossart et al. (2018). Briefly, samples were incubated in 120 µl of 2% sodium dodecyl sulfate (SDS) for 24 h at room temperature. The BCA assay was undertaken according to the manufacturer’s protocol (Thermo Scientific, Waltham, MA, USA).

### 2.7 Human Gingival Fibroblast Culture

#### 2.7.1 Cell Line and Culture Media

Human gingival fibroblasts were isolated from healthy donors and kindly donated by Bios Laboratory at Reims University. Human gingival fibroblasts (HGF) in passaging <10 were cultured in Dulbecco’s modified Eagle’s medium-high glucose (DMEM) supplemented with 10% fetal bovine serum (Biosera, Shanghai, China), 1% penicillin–streptomycin (Gibco, Grand Island, NY, USA) and 1% sodium bicarbonate (Gibco) (complete medium).

#### 2.7.2 Cell Cytotoxicity

(PLL_30_-Fn)_10_ films, Fn monolayers, PLL_30_ monolayers, and uncoated glass samples were prepared in sterile conditions. A total of 5,000 cells/cm^2^ were seeded under standard cell culture conditions (complete medium) and incubated at 37°C and 5% CO_2_ during 48 h. Cellular cytotoxicity was determined using the Pierce LDH cytotoxicity assay (Thermo Fisher Scientific) following the manufacturer’s protocol. Released LDH activity into the culture media was calculated by reading the absorbance of the samples at 490 nm.

#### 2.7.3 Metabolic Activity

The metabolic activity of HGF was analyzed using the WST-1 colorimetric assay. A total of 5,000 cells/cm^2^ were seeded on sterile substrates using complete medium and incubated at 37°C and 5% CO_2_. After 2, 4, and 7 days, respectively, cells were washed with PBS (2×) and incubated in fresh medium with 10% WST-1 reagent (Sigma, St. Louis, MO, USA) at 37°C and 5% CO_2_ for 30 min. The absorbance was measured at 440 nm, to determine the amount of formazan dye formed that is directly proportional to the amount of mitochondrial dehydrogenase into the given culture.

#### 2.7.4 Cell Proliferation/DNA Mass Quantification

DNA content was measured using CyQUANT Cell Proliferation Assay (Invitrogen, Carlsbad, CA, USA). Dried substrates were frozen at −80°C and subsequently thawed at room temperature. A volume of 200 μl of the CyQUANT® GR dye/cell-lysis buffer, prepared following the manufacturer’s protocol, was added to the samples and incubated at room temperature for 5 min protected from light. The fluorescence of the supernatant was measured at 485 nm.

#### 2.7.5 Cell Morphology

A total of 5,000 cells/cm^2^ were seeded on sterile substrates using complete medium and incubated at 37°C and 5% CO_2_ during 4 h. Cells were fixed with a 4% (w/v) p-formaldehyde solution in PBS (Sigma) for 10 min at room temperature and permeabilized with 0.2% (v/v) Triton X-100 (Sigma) for 15 min. Non-specific binding sites were blocked with a 0.5% bovine serum albumin (BSA) solution in PBS for 30 min. The nuclei of cells were revealed by incubating the samples with 0.5 μg/ml 4,6-diamidino-2-phenylindole dihydrochloride (DAPI, Sigma) diluted in 0.5% BSA in PBS for 1 h at room temperature. The cytoskeleton of cells was visualized with a 5-μg/ml phalloidin-FITC (Sigma) solution in 0.5% BSA in PBS for 45 min. Focal adhesions of cells were analyzed by incubating the samples with 100 μg/ml mouse polyclonal anti-vinculin antibody (Sigma) dissolved in 0.5% BSA in PBS. Specifically, bound antibodies were revealed incubating the samples with 100 μg/ml TRITC-coupled secondary antibodies (Sigma) dissolved in 0.5% BSA in PBS for 1 h. Samples were washed with PBS, mounted in ProLong™ Gold (Invitrogen), and examined using a LSM710 confocal microscope (Carl Zeiss).

#### 2.7.6 Fn Reorganization

To analyze the reorganization of Fn within the films, substrates were built using fluorescently labeled Fn (Fn*) with Alexa Fluor 568. A total of 5,000 HGF/cm^2^ were seeded on the samples using complete medium and incubated at 37°C and 5% CO_2_ during 2, 4, and 7 days, respectively, and immunostained as previously described (Section 2.7.5). For STED microscopy experiments, mouse monoclonal anti-vinculin V9131 (Sigma-Aldrich) diluted 1:400 in PBT (PBS + 0.1% Tween 20 + 2% BSA) was used to control focal adhesion formation, and the samples were incubated for 45 min at room temperature. Mouse Abberior STAR RED (Abberior, Göttingen, Germany) diluted 1:200 in PBT was added to the samples as secondary antibody and incubated for 1 h at room temperature. Substrates were washed with PBS (3×) for 15 min and mounted on microscope slides using Abberior Mount Liquid. Samples were examined using Abberior STEDYCON attached to a Zeiss Axio Imager.

### 2.8 Antibacterial Properties

#### 2.8.1 Bacterial Strains and Growth Conditions

*Staphylococcus aureus* (CIP 4.83) and *Staphylococcus epidermidis* (CIP 105.777) were obtained from the “Collection de l’Institut Pasteur Paris” and *Streptococcus mutans* (DSM 20523) was obtained from the DSMZ-German collection. Bacterial cultures were stored in Tryptic Soy Broth (TSB, BD™ Biosciences, San Jose, CA, USA) containing 20% glycerol at −20°C. Prior to use in adherence experiments, bacterial suspensions were prepared in sterile TSB and grown overnight at 37°C and 200 rpm.

#### 2.8.2 Bacterial Adhesion

The optical density (OD) of the *S. aureus*, *S. epidermidis*, and *S. mutans* suspensions was measured with a spectrophotometer at λ = 600 nm and the concentration adjusted to 10^6^ CFU/ml in all cases. A volume of 1 ml of bacterial suspension in PBS was added to the sterile substrates and incubated at 37°C for 2 h. Non-adherent cells were removed by rinsing the substrates (2×) with 0.9% (w/v) NaCl solution. Adherent cells were recovered by sonication in 0.5 ml (2×) 0.9% (w/v) NaCl solution, and the supernatant was serially diluted 1:10 to obtain concentrations of 10°, 10^–1^, and 10^–2^ CFU/ml, respectively. A volume of 100 µl of each dilution was pipetted, spread on agar plates (Tryptic Soy Agar, TSA), and incubated at 37°C for 16–48 h. The viable number of bacteria was estimated within a countable range (30–300 CFU per plate). For LIVE/DEAD experiments, *Bac*Light™ Bacterial Viability Kit (Thermo Fisher Scientific) was employed. *Bac*Light solution (3 μl) was diluted into 1 ml of Milli-Q water and added to the samples for 20 min protected from light. The substrates were washed with Milli-Q water and mounted in ProLong™ Gold (Invitrogen). Attached bacteria were viewed using a LSM710 confocal microscope (Carl Zeiss), with live and dead cells visualized in green and red, respectively.

### 2.9 Statistical Analysis

Statistical analysis was performed using GraphPad Prism software (San Diego, CA, USA), and an analysis of variance (ANOVA) or independent samples t-test was used. Where appropriate, the statistical significance was reported for *p* < 0.05. All experiments were repeated in triplicate with three technical replicates for each experimental value.

## 3 Results

### 3.1 Thin Film Properties

#### 3.1.1 LbL Film Assembly

**FIGURE 1:**
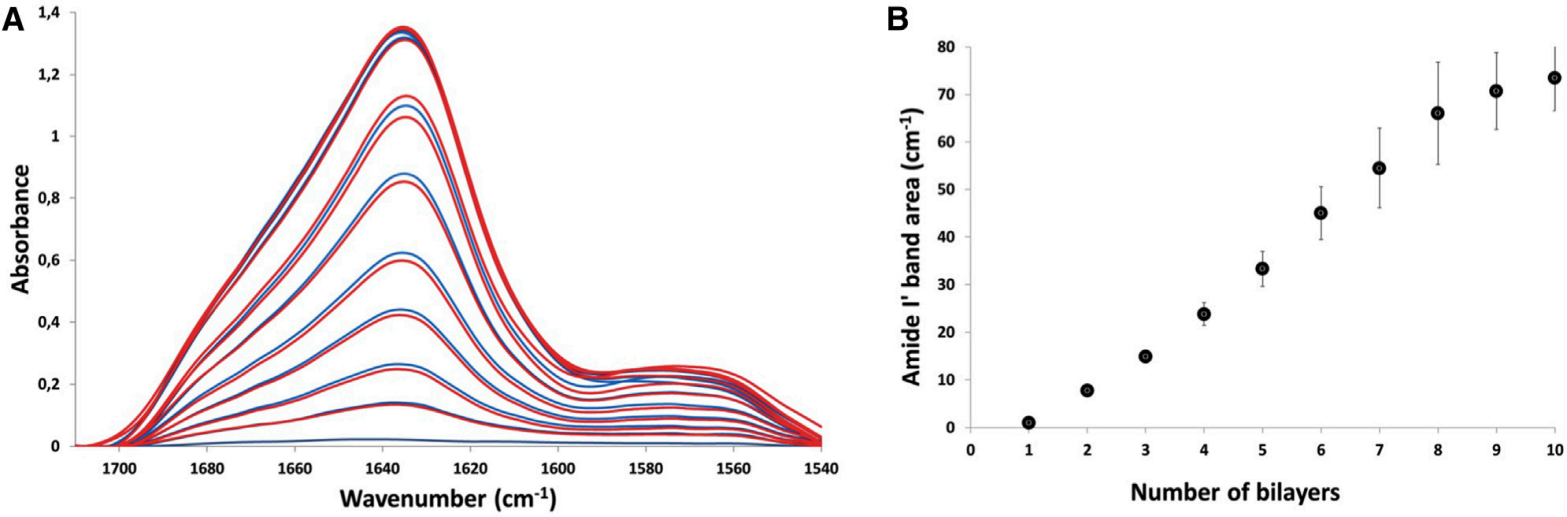
Layer-by-layer film assembly followed by ATR-FTIR: **(A)** ATR-FTIR spectra acquired during the buildup of a (PLL_30_-Fn)_10_ LbL thin film, after each adsorption of PLL_30_ (blue) and after each adsorption of Fn (red). The film was constructed on a silicon crystal. The amide I′ band (1,600–1,700 cm^−1^) and amide II′ band (1,540–1,600 cm^−1^) are represented. **(B)** Evolution of the Amide I′ band area measured by ATR-FTIR during multilayer film formation. The amide I′ band area measured after each PLL_30_ adsorption is shown. The vertical lines represent the estimated error on each point.

Thin film assembly was assessed by attenuated total reflection-Fourier transform infrared (ATR-FTIR) spectroscopy (Müller et al., 1998). Figure 1A shows the evolution of the infrared spectra corresponding to the sequential adsorption of PLL_30_ and Fn at 0.5 mg/ml in deuterated phosphate buffer or deuterated water, on silicon wafer. Similar results were obtained in both buffers, suggesting that the presence of phosphate ions has no influence on film assembly. As previously described, it is possible to follow the alternate deposition of PLL and Fn and subsequently the film construction following the Amide I′ band (1,610–1,700 cm^−1^) corresponding to the peptide carbonyl stretching modes ν(CO) (Gand et al., 2017). The intensity of the Amide I′ band increases with the number of layers. However, the absorbance related to Fn adsorption is significantly higher compared to PLL_30_, suggesting high adsorption of Fn with low adsorption of PLL_30_. This result proves that low molecular weight PLL does not compromise thin film assembly. When plotted against the number of layers, the integrated area of the amide I′ band increases. However, whereas film growth appears linear from 2 to 7 bilayers, its progression starts to diminish after 8 bilayers, with a significant decrease in incremental adsorption. After 10 bilayers, a plateau is reached (Figure 1B). PLL_30_-Fn LbL assembly results in sub-linear, saturating growth, as previously observed with high molecular weight (HMW) PLL (70–150 kDa). If we compare the Amide I′ band area of films constructed with PLL_30_ or PLL_70-150_ at the same concentration of 0.5 mg/ml, we can observe higher values when films are built with PLL_30_ (70 cm^−1^) than with high molecular weight PLL (40 cm^−1^). Moreover, with the high molecular weight PLL, the film stalls clearly after a maximum of 5 bilayers, suggesting higher Fn and PLL adsorption when a low molecular weight PLL is used (Supplementary Figure S1). As the film stops growing after 10 bilayers, the following experiments were performed on (PLL_30_-Fn)_10_ films built on glass slides. The quantity of total protein adsorbed on glass substrates in (PLL_30_-Fn)_10_ was assessed by BCA assay, and the results show a protein adsorption of 16 μg/cm^2^.

#### 3.1.2 Surface Morphology and Thickness

**FIGURE 2:**
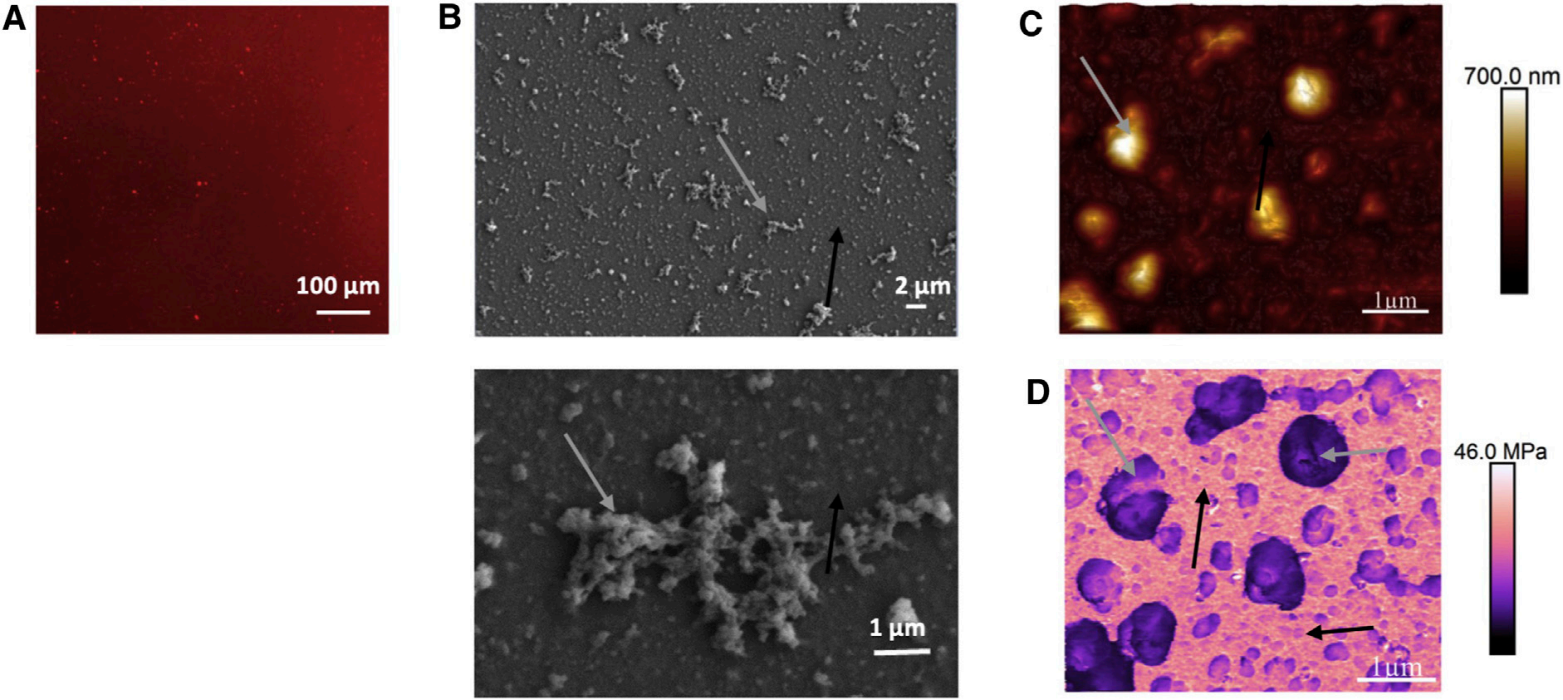
Fluorescent microscopy **(A)**, scanning electron microscopy **(B)**, and atomic force microscopy **(C)** representative images showing (PLL_30_-Fn)_10_ thin film surface organization and morphology. **(D)** Young’s modulus measurement of (PLL_30_-Fn)_10_ thin films obtained by AFM. For **(A),** Fn is fluorescently labeled with Alexa Fluor 568.

The organization and characterization of the (PLL_30_-Fn)_10_ film were evaluated based on images from fibronectin fluorescent labeling (Figure 2A), scanning electron microscopy (Figure 2B), and atomic force microscopy (Figure 2C). Images show that the surface of the glass substrate is completely covered by the film. At a large scale, a complete and consistent distribution of Fn in all the film dimensions is evident (Figure 2A). However, whereas the surface is totally covered, the organization of the molecular polymer and the film appears heterogeneous at a smaller scale, with the presence of aggregates of different sizes, ranging from 1 to 10 µm. The same observations can be made with atomic force microscopy (AFM) images. The presence of these aggregates, and the heterogeneity in their distribution and organization, renders difficult the calculation of the roughness of the surface. Considering zones without large aggregates, it is possible using AFM images to estimate film thickness. An average thickness ranging from 200 to 300 nm is found, which is very thin. However, the thickness can go up to 700 nm to 1 µm in zones containing large aggregates. Some rigidity parameters of elements constituting the films can be estimated with AFM measurement of the Young’s modulus (Figure 2D). The images obtained exhibit high rigidity of the homogeneous film distributed around the aggregates (up to 46 MPa Figure 2D, black arrows), whereas aggregates show lower rigidity values (5–30 kDa Figure 2D, gray arrows). Such large structures were already observed with HMW PLL and could result from molecule self-assembly (protein or polyelectrolyte polymers) (Gand et al., 2017). The Young’s modulus values obtained are in accordance with values of protein aggregates that could range from 15 to 30 MPa (Lam and Ikeda 2017) which is lower than values obtained when proteins are self-assembled into fibrils (GPa) (Gilbert et al., 2017).

### 3.2 Primary Gingival Fibroblast Behavior on PLL_30_-Fn Thin Films

#### 3.2.1 Cell Morphology and Thin Film Cytotoxicity

**FIGURE 3:**
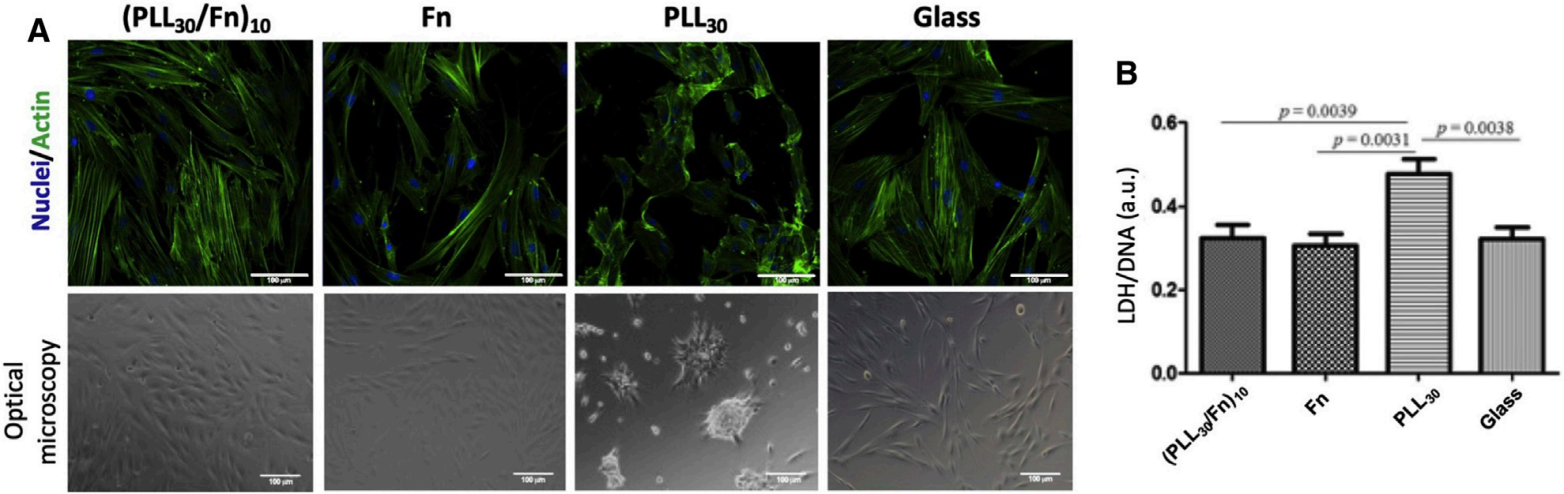
Morphology and cytotoxicity of human gingival fibroblasts (HGF) on (PLL_30_-Fn)_10_ films, Fn monolayer, PLL_30_ coating, and glass coverslip after 48 h of incubation. **(A)** Morphology observations of HGF stained for nuclei (DAPI—blue) and actin (Phalloidin—green) on the first line. Micrographs of optical microscopy of HGF on the second line. Typical fibroblastic morphology on (PLL_30_-Fn)_10_ films compared to positive control (glass). PLL_30_ coating shows a stressful environment for cells. Scale bar = 100 µm. **(B)** Cell cytotoxicity obtained by LDH assay. Results were normalized with DNA quantification (CyQUANT™). (PLL_30_-Fn)_10_ films are non-cytotoxic compared to positive control (glass). Statistically different at *p* < 0.05.

To explore the potential of (PLL_30_-Fn)_10_ thin films as proadhesive coatings on percutaneous permucosal implants, HGF behavior was investigated. The morphology of HGF after 48 h of incubation was observed by confocal and optical microscopy (Figure 3A). In contact with the (PLL_30_-Fn)_10_ film, fibroblasts show a typical elongated morphology with abundant actin filaments. Similar results were observed on the Fn monolayer and glass control. In contrast, on PLL_30_ coating cells are round-shaped or assembled in circular aggregates, which suggests that the PLL monolayer constitutes a stressful environment. In addition, LDH assay was done to evaluate the cytotoxicity of the (PLL_30_-Fn)_10_ film. Results are expressed by normalizing the LDH released by the quantified DNA (Figure 3B). LDH/DNA values of the (PLL_30_-Fn)_10_ film, Fn monolayer, and glass are similar, which demonstrates that all these coatings are non-cytotoxic. In comparison, the LDH/DNA value of PLL_30_ coating is higher and statistically different (*p* < 0.05), which confirms its cytotoxicity.

#### 3.2.2 Cell Proliferation and Metabolic Activity

**FIGURE 4:**
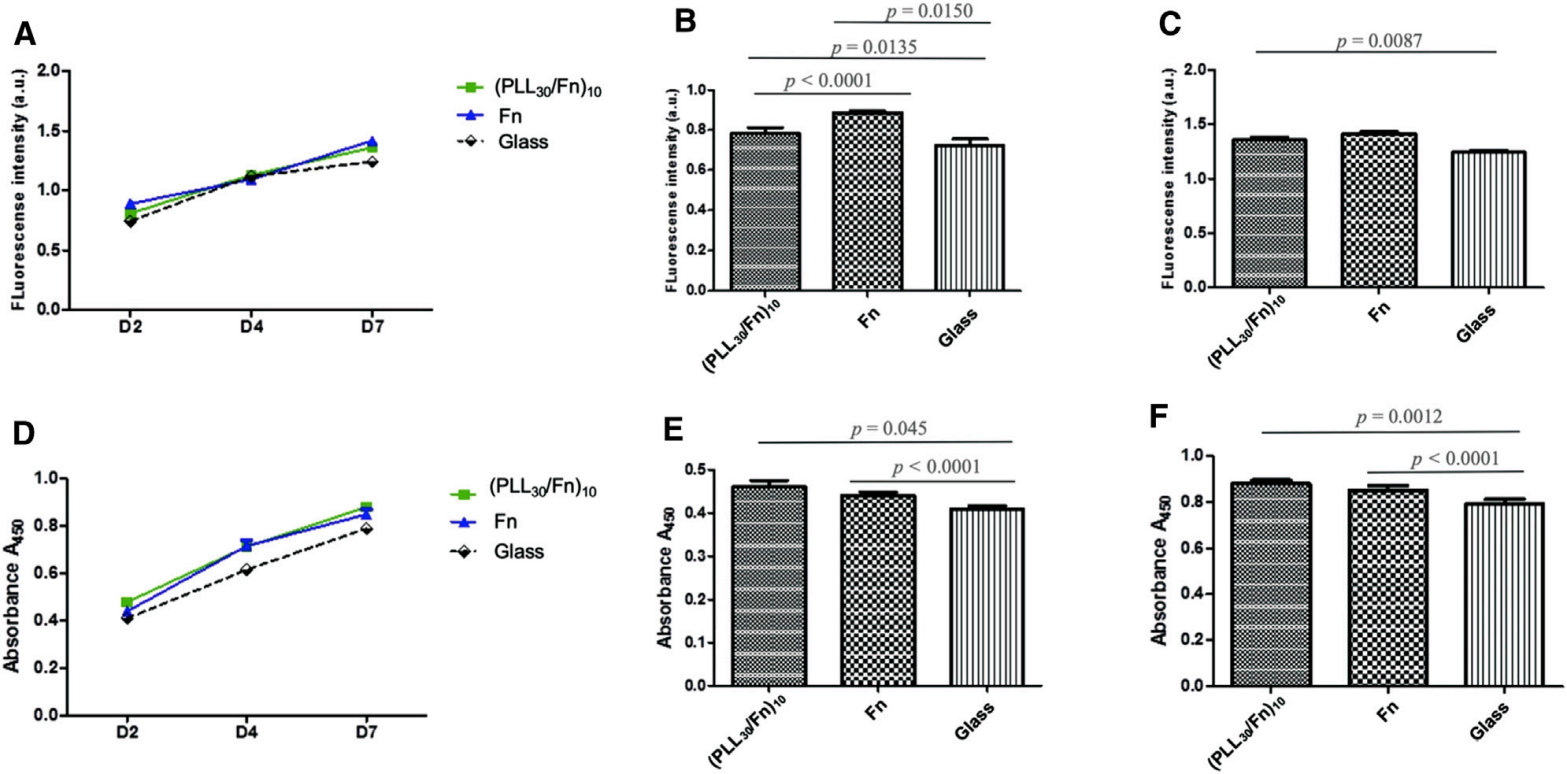
Proliferation and metabolic activity of human gingival fibroblasts (HGF) on (PLL_30_-Fn)_10_ films, Fn monolayer, and glass coverslip. **(A)** CyQUANT™ results of cell proliferation of HGF at 2, 4, and 7 days of incubation. Histograms representing more in detail cell proliferation at 2 days **(B)** and 7 days **(C)** of incubation. **(D)** WST-1 quantitation of metabolic activity of HGF on different conditions over time. Histograms representing more in detail metabolic activity corresponding to 2 days **(E)** and 7 days **(F)** of incubation. Statistically different at *p* < 0.05.

HGF cell behavior was assessed by cell proliferation (Figures 4A–C) and metabolic activity (Figures 4D–F) at 2, 4, and 7 days of incubation. As PLL_30_ coating demonstrated cytotoxicity, cell behavior was evaluated only in (PLL_30_-Fn)_10_ films and the Fn monolayer. Figures 4A,D show the HGF proliferation kinetic obtained by DNA quantification (CyQUANT™) and metabolic activity of HGF obtained by WST-1 assay, respectively. An increase in cell proliferation (Figure 4A) and metabolic activity (Figure 4D) is observed in each condition. Figures 4B,C focus on the results obtained on day 2 and day 7 respectively and show in a more detailed fashion the values of fluorescence intensity of cell proliferation. Two days after cell seeding, cell proliferation is significantly higher for (PLL_30_-Fn)_10_ films compared to glass control and the same trend is observed 7 days post-seeding. Yet there is no significant difference in proliferation between (PLL_30_-Fn)_10_ films and the Fn monolayer at 7 days of incubation. The metabolic activity (Figures 4E,F) revealed a similar tendency at 2 and 7 days; thus, the increase in metabolic activity is directly related to cell proliferation.

#### 3.2.3 Cell Adhesion

**FIGURE 5:**
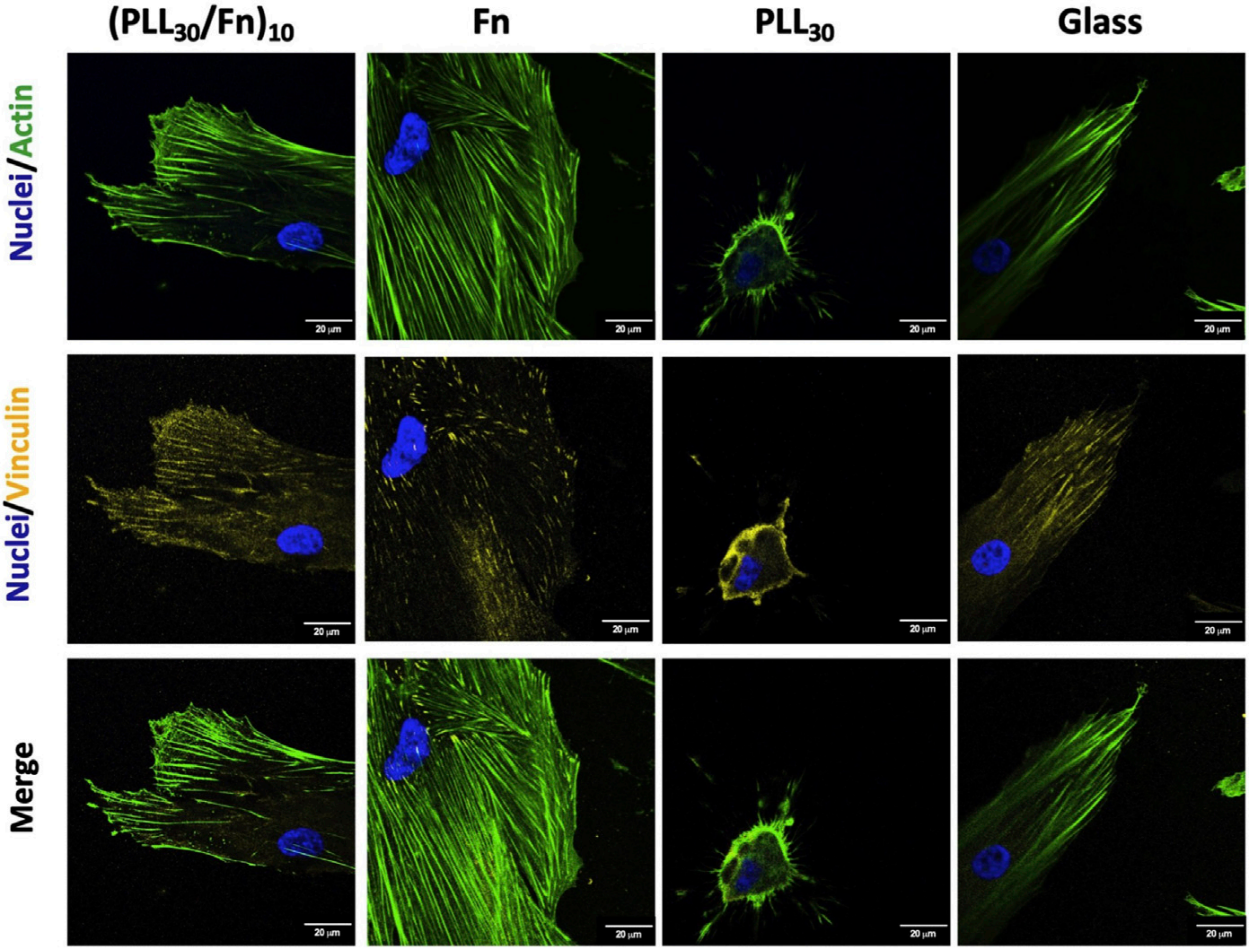
Representative fluorescence microscopy images of cell adhesion of human gingival fibroblasts (HGF) on (PLL_30_-Fn)_10_ films, Fn monolayer, PLL_30_ coating, and glass coverslip analyzed by immunostaining: nuclei (DAPI-blue), actin (phalloidin-green), and vinculin (TRITC-yellow) after 4 h of incubation. Scale bar = 20 μm.

Adhesion of HGF on (PLL_30_-Fn)_10_ films and the Fn monolayer was evaluated at 4 h of incubation by immunostaining. Cells were stained for cellular structures as nucleus, actin, and vinculin and visualized by confocal microscopy (Figure 5). Vinculin is a component of focal adhesion, and the presence of this molecule would give more information about the cell–matrix interaction. In (PLL_30_-Fn)_10_ films, Fn monolayer, and glass control, cells present abundant actin fibers arranged in bundles. Likewise, lamellipodia formation is more prominent in (PLL_30_-Fn)_10_ films compared to the Fn monolayer. Vinculin is mainly distributed at the cytoplasm on the Fn monolayer whereas in the case of (PLL_30_-Fn)_10_ films and glass control, vinculin is present not only at the cytoplasm but also in majority at the cell periphery. Therefore, an increase in the vinculin beams was remarkable in (PLL_30_-Fn)_10_ films compared to the Fn monolayer. These results strongly suggest a better cell–matrix interaction between HGF and (PLL_30_-Fn)_10_ films.

#### 3.2.4 (PLL_30_-Fn)_10_ Film Remodeling

**FIGURE 6:**
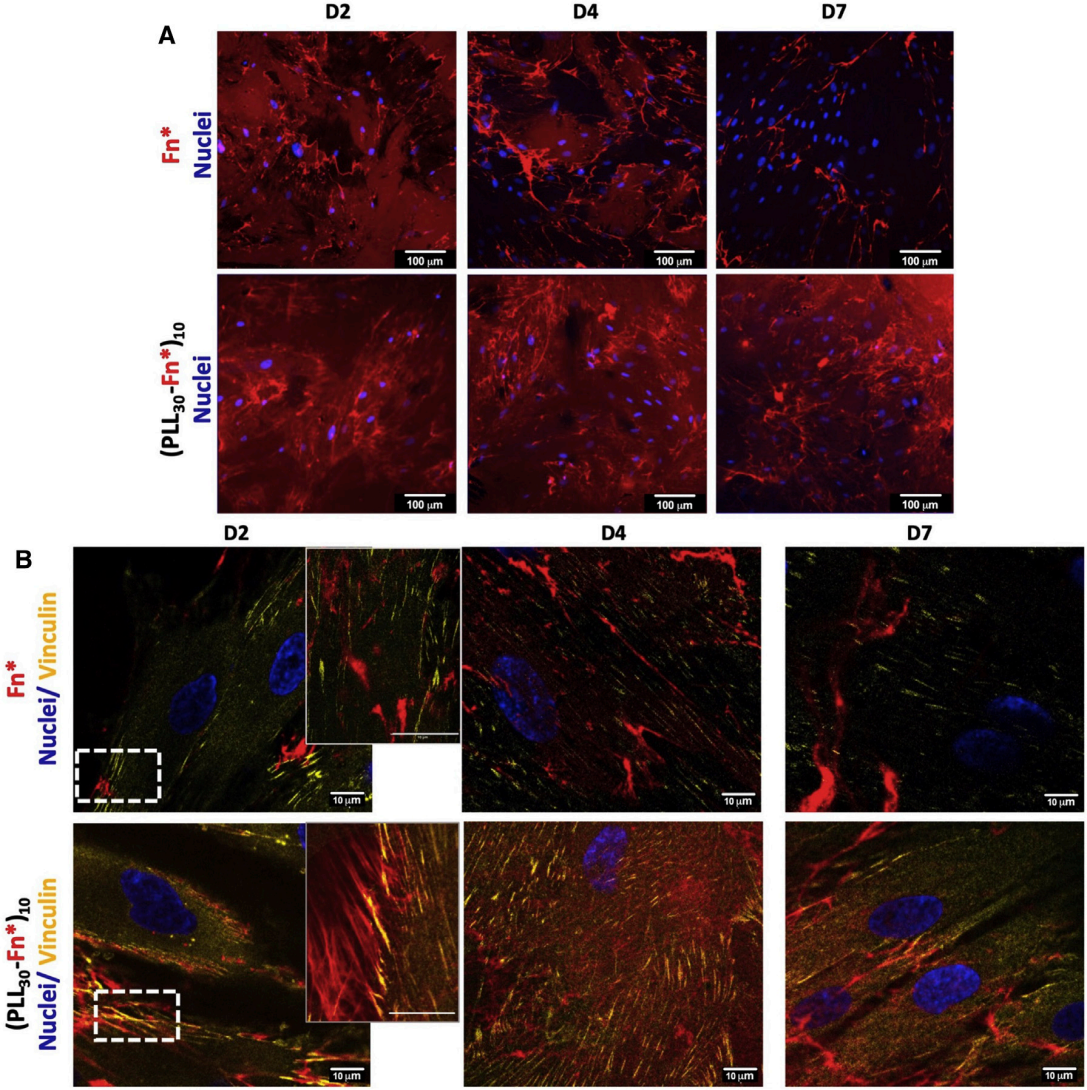
**(A)** Fn reorganization by human gingival fibroblasts (HGF) on the Fn monolayer and (PLL_30_-Fn)_10_ films. Cells were cultured for 2, 4, and 7 days and stained for nuclei (DAPI—blue). (PLL_30_-Fn)_10_ films exhibit an increase in Fn fibril formation over time. Scale bar = 100 μm. **(B)** Distribution of focal adhesion sites of HGF cells on Fn monolayer and (PLL_30_-Fn)_10_ films analyzed by immunostaining of vinculin (Star Red—yellow) and nuclei (DAPI—blue). The second column corresponds to magnified images of the white rectangles of the first column. Vinculin is particularly linked to Fn fibrils in (PLL_30_-Fn)_10_ films. Scale bar = 10 μm. Fn was previously labeled with Alexa Fluor 568 for both analyses.

Fn reorganization within (PLL_30_-Fn)_10_ films and the Fn monolayer by HGF was observed by confocal microscopy using fluorescently labeled Fn during film assembly. A complete and homogeneous distribution of Fn (red) on the glass substrate was observed for (PLL_30_-Fn)_10_ thin films and the Fn monolayer (Supplementary Figure S2). Fn is reorganized into fibrils in both (PLL_30_-Fn)_10_ films and the Fn monolayer after 2 days of cell culture (Figure 6A). As previously described (Gand et al., 2017), a decrease in non-assembled Fn was demonstrated after 4 days of cell culture until complete rearrangement in fibrils at 7 days for the Fn monolayer. In comparison, the quantity of the remaining non-assembled Fn after 7 days of cell culture is significantly higher in (PLL_30_-Fn)_10_ films with the presence of Fn fibrils. (PLL_30_-Fn)_10_ films could thus provide a long-term reservoir of Fn. Formation of Fn fibrils by HGF demonstrates the presence of focal adhesions and the direct interaction between intracellular components and extracellular support (Pompe et al., 2005).

To better understand the cell–matrix interaction between HGF and extracellular Fn, focal adhesions were evaluated by vinculin immunostaining. Figure 6B shows the interaction of vinculin and Fn in (PLL_30_-Fn)_10_ films and the Fn monolayer. The focal adhesion is significantly higher in (PLL_30_-Fn)_10_ films compared to the Fn monolayer (Figure 6B). Interestingly, focal adhesions co-localized with Fn fibers and seemed to follow Fn fibril orientation. This behavior was not observed in the Fn monolayer despite the presence of Fn fibrils and could explain the decrease in vinculin signal after 7 days of cell culture. These images suggest a focal adhesion pattern between vinculin and Fn fibrils in the synthetic matrix.

### 3.3 Antibacterial Effect of (PLL_30_-Fn)_10_ Thin Films

**FIGURE 7:**
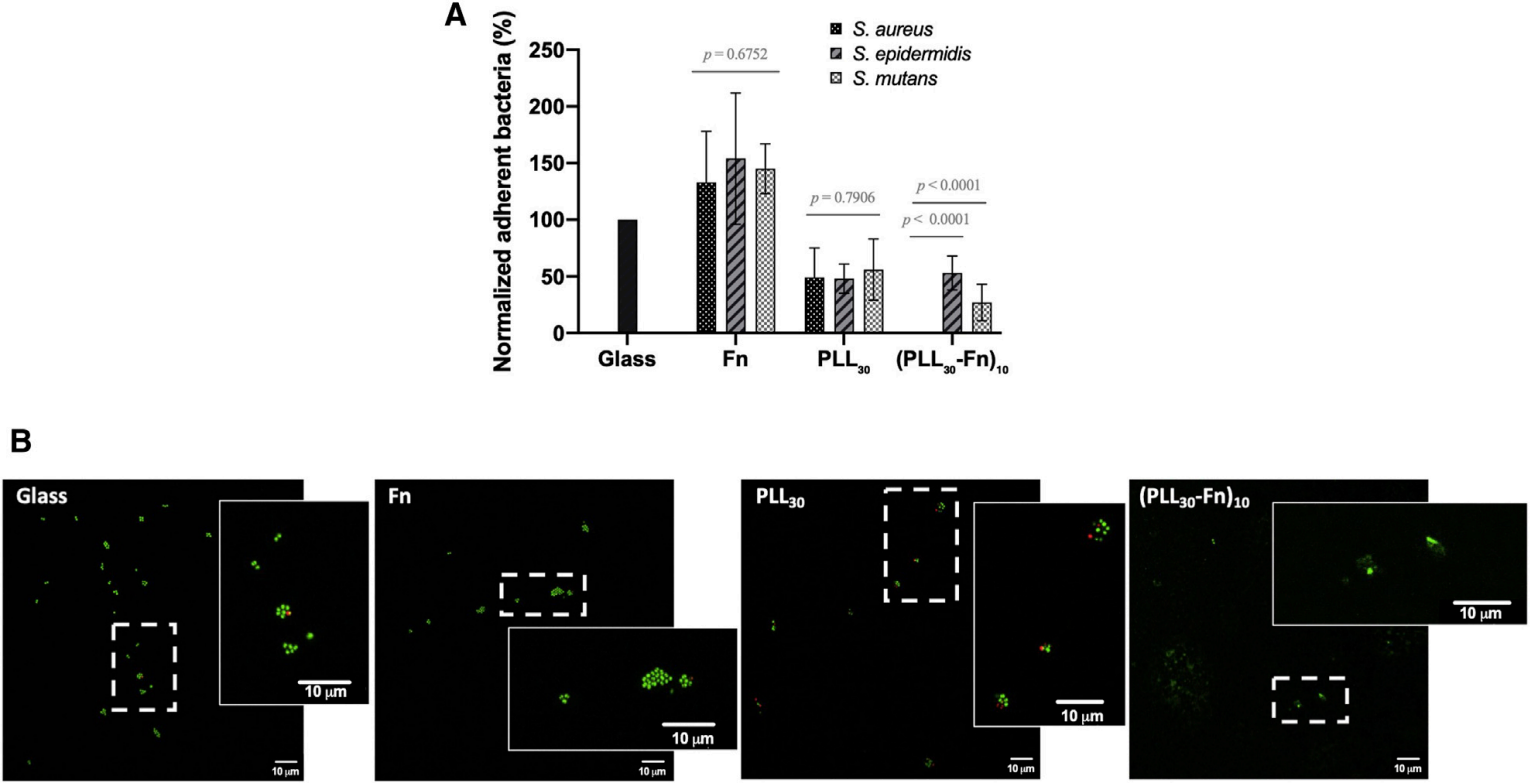
Bacterial attachment on glass, Fn monolayer, PLL_30_ monolayer, and (PLL_30_-Fn)_10_ thin films after 2 h of incubation. **(A)** Percentage of adherent *S. aureus, S. epidermidis*, and *S. mutans* on Fn monolayer, PLL_30_ monolayer, and (PLL_30_-Fn)_10_ films normalized to bacteria adhered on glass (*n* = 7). No *S. aureus* are found to adhere to (PLL_30_-Fn)_10_ thin films. Data are normalized for each strain to their respective glass control mean. Error bars indicate the standard deviation on the mean normalized data. **(B)** Representative images of LIVE/DEAD staining of adherent *S. aureus* on glass, Fn monolayer, PLL_30_ monolayer, and (PLL_30_-Fn)_10_ thin films (*n* = 3). The magnified captions correspond to the area surrounded by dashed rectangles in the original images. Note: Green indicates live bacteria, red indicates dead bacteria. Scale bar = 10 μm. Statistically different at *p* < 0.05.

We tested the adhesion of three bacterial strains *S. aureus*, *S. epidermidis*, and *S. mutans*, on glass, Fn or PLL_30_ coatings, and (PLL_30_-Fn)_10_ thin film. The number of adherent bacteria after 2 h was determined, and results are normalized to bacterial adhesion on glass slides (Figure 7A). First, we observe that bacterial adhesion is increased to about 140% for the three strains on Fn coatings. This result was expected as it has been reported that bacteria possess adhesive molecules able to recognize immobilized Fn. *S. epidermidis* adhere to Fn *via* teichoic acid, an essential component of the *S. epidermidis* cell wall (Hussain et al., 2001). *S. aureus* and *S. mutans* adhesion to Fn is mediated by FnBPA/B and SmFnB, respectively, Fn binding proteins, known as surface adhesin proteins from the MSCRAMM family (microbial surface components recognizing adhesive matrix molecules) (Foster and Höök 1998; Chia et al., 2000; Mongodin et al., 2002). PLL_30_ monolayer coating diminishes bacterial adhesion to 40%–50%, which is consistent with previous studies where PLL has been shown to have antibacterial effects in solution or coated on surfaces (Conte et al., 2007; Colville et al., 2010; Dubois et al., 2013). The association of the two molecules forming a polyelectrolyte multilayer film (PLL_30_-Fn)_10_ leads to a similar adhesion of *S. mutans* and *S. epidermidis* compared to the PLL_30_ monolayer, but surprisingly, (PLL_30_-Fn)_10_ totally inhibits *S. aureus* adhesion to the surface. In order to confirm these results, the different samples were stained with LIVE/DEAD dyes to visualize adherent bacteria. Figure 7B shows LIVE/DEAD staining of *S. aureus* on glass, Fn or PLL_30_ coatings, and (PLL_30_-Fn)_10_ thin film. On glass slides, *S. aureus* adhere and tend to form cluster in grapes with the presence of very few dead bacteria. A similar behavior is observed on Fn coating, but in this case, the number of adherent bacteria is higher, bacteria in division are visible, and there are no dead cells on the surface. On the contrary, on PLL_30_ coating, bacteria appear more isolated and the number of adherent cells is clearly diminished compared to glass or Fn coating with an increase in dead bacteria. Finally, on (PLL_30_-Fn)_10_ thin film, no colony is observed on the entire surface, and only bacterial debris remained, confirming the strong anti-adhesive properties of the modified surface. The (PLL_30_-Fn)_10_ thin film prevents bacterial adhesion of *S. epidermidis* and *S. mutans* but to a lesser extent than what is observed with *S. aureus* (Supplementary Figure S3).

## 4 Discussion and Conclusion

One of the main challenges in biomaterial implantation is the correct bio-integration that conditions the successful performance of the implant in the human body. In the context of percutaneous permucosal prosthesis, like dental implants or ITAP, implantation is often compromised by loose soft tissue–implant interaction and subsequent bacterial infection. The success of biomaterial integration is conditioned by the type of cells that will first attach to the surface: eukaryotic cells have to adhere primarily to bacterial adhesion, which constitutes a “race to the surface.” There is therefore a crucial need to improve implant–host tissue cross talk by developing interfacial strategies that will enhance soft-tissue sealing and prevent bacterial adhesion.

In the last decades, there has been increasing interest in the functionalization of surfaces with Fn, by direct adsorption or grafting on different surfaces, to enhance cell adhesion, proliferation, and spreading (Toworfe et al., 2004; Middleton et al., 2007; Daum et al., 2020). In particular, Ghadhab et al. (2021) showed that Ti surfaces functionalized with covalently grafted Fn enhance dermal fibroblast adhesion, spreading, and proliferation and that the adhesion strength between the functionalized material and a bioengineered dermal tissue is higher (Ghadhab et al., 2021). Recently, studies focused on the use of Fn in combination with other molecules in order to create 3D biomimetic environments to enhance cell attachment (Ngankam et al., 2004; Zanina et al., 2011) and cytocompatibility for tissue engineering (Ravi et al., 2013; Mauquoy and Dupont-Gillain 2016).

Bacterial adhesion to the implant surface constitutes the first step to biofilm formation at the implantation site. This can lead to infections that can induce implant failure and in the worst case to amputation or death. It is thus of primary interest to find material/interfaces with anti-adhesive properties. Surface contamination occurs mainly during surgery and comes from the environment or from the patient himself (from the skin and gut microbiote, or from human blood microbiote) (Ribeiro et al., 2012). To prevent postimplantation infections, antibiotics are systematically used in prophylaxis, but in the last decades antibiotic-resistant strains had emerged and constitute a major concern for the World Health Organization (Tacconelli et al., 2018). Alternative strategies using natural molecules or polypeptides are therefore finding growing interests.

Based on these findings, we developed a bifunctional coating that favors cell adhesion and prevents bacterial attachment, combining Fn and low molecular weight PLL in an LbL architecture. Here we show that it is possible to create an interface enriched in Fn, biomimetic of the ECM, *via* direct incorporation of Fn during LbL assembly. This is of particular interest as deposition of Fn on a surface leads to a monolayer with a limited adsorption (∼250 ng/cm^2^ on a silica surface) (Baujard-Lamotte et al., 2008) and Fn adsorption on top of an Fn monolayer is impossible (Gand et al., 2017) whereas a high quantity of Fn, around 16 μg/cm^2^, is reached in (PLL_30_-Fn)_10_ thin films. Hence, thin films could constitute a reservoir for Fn and PLL molecules. PLL has been chosen to bring the antibacterial properties as cationic polymers have been shown to disrupt the bacterial membrane leading to the leakage of intracellular substances and therefore to the death of bacteria (Carmona-Ribeiro and de Melo Carrasco, 2013; Huang et al., 2016). Recent studies have shown that LbL thin films containing polycationic polymers exhibit antimicrobial properties and that these properties are driven by the polycation molecular weight. Mutschler et al. have shown that low molecular weight poly-arginine (PAR_30_, 30 residues) associated with hyaluronic acid (HA) in thin coatings displays high antibacterial activity compared to high molecular weight PAR (PAR_100_ or PAR_200_), due to the ability of PAR_30_ to diffuse at the surface of the film and to interact with bacteria (Mutschler et al., 2016). Similar results were obtained by Alkekhia D. and Anita Shukla A.: thin films built with the combination of PLL and HA inhibited 60%–70% *S. aureus* attachment compared to the uncoated surface. Anti-adhesive properties are brought by HA while PLL is suggested to inhibit bacterial growth (Alkekhia and Shukla 2019). As PLL-Fn thin films built with high molecular PLL (70–150 kDa) showed no antibacterial properties, we decided to use a low molecular weight PLL composed of 30 residues. PLL_30_ simple coating constitutes a cytotoxic, stressful environment for HGF, but in association with Fn, this negative effect totally disappears and (PLL_30_-Fn)_10_ thin films enhance HGF adhesion and proliferation compared to glass slides. Nevertheless, in future studies, it could be interesting to explore other structures of PLL, like dendrigraft PLL, or DGLs, known to present lower cytotoxicity while exhibiting high antibacterial activity (Francoïa and Vial 2018). HGF on (PLL_30_-Fn)_10_ thin films present a higher amount of focal adhesion compared to glass or Fn coatings, suggesting stronger adhesion of cells to the surface. Focal adhesion generates traction force that will participate in the remodeling of Fn within the thin film. If Fn conformation and integrin-binding site accessibility play a key role in early cell adhesion, Lin et al. have shown that protein reorganization carried out by cells enhances long-term cell adhesion and that protein adsorption force on a biomaterial is directly related to surface chemistry (Lin et al., 2015).

Finally, (PLL_30_-Fn)_10_ thin films prevent *S. aureus* bacterial initial attachment and significantly reduce *S. epidermidis* and *S. mutans* adhesion compared to bare surfaces. The impact on *S. aureus* adhesion is also stronger on (PLL_30_-Fn)_10_ thin films compared to the PLL_30_ monolayer, showing that thin films constitute a reservoir for the delivery of PLL_30_. Bacterial adhesion being the initial step toward biofilm formation on biomaterials, prevention of bacterial attachment could have a strong impact on implant-related infections. The potential of (PLL_30_-Fn)_10_ films to limit biofilm development of the three bacteria will be investigated any further in coculture with HGF.

These findings shed light on the potential of thin films, constructed with Fn and PLL_30_, to enhance cell adhesion and favor material-tissue integration while avoiding bacterial attachment.

## Data Availability

The original contributions presented in the study are included in the article/Supplementary Material; further inquiries can be directed to the corresponding authors.
