# Supplementary material for: Poly-L-Lysine and Human Plasmatic Fibronectin Films as Proactive Coatings to Improve Implant Biointegration
Source: Front Bioeng Biotechnol. 2022 Jan 17;9:807697. doi: 10.3389/fbioe.2021.807697 (PMC8801876; doi:10.3389/fbioe.2021.807697)
Supplement: Supplementary file 1 [file DataSheet1.docx]

**Supplementary Data**

**
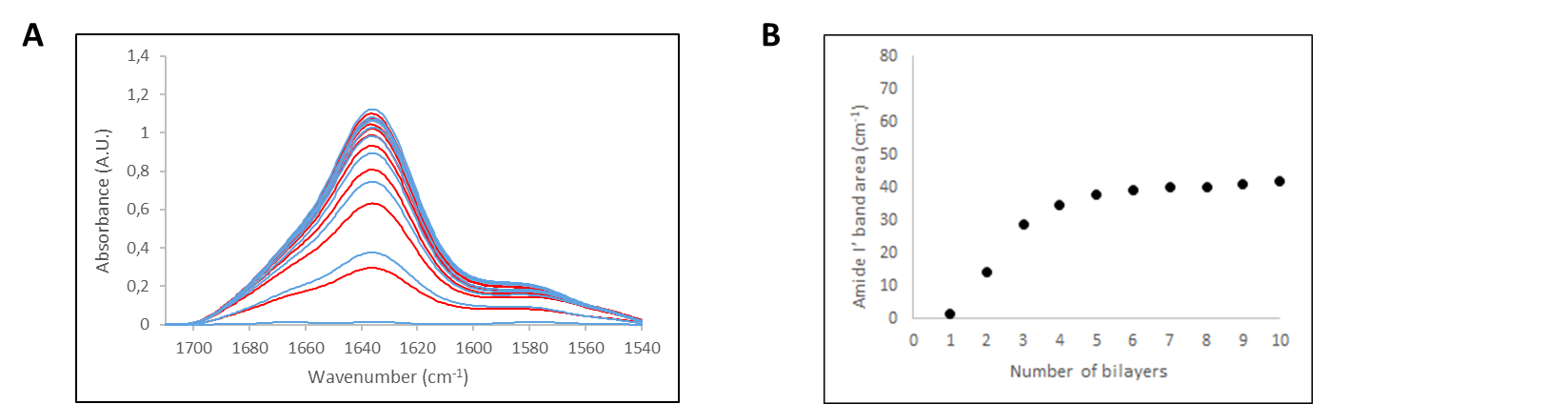
**

**Figure S1:** Layer-by-Layer film assembly followed by ATR-FTIR. (A) ATR-FTIR spectra acquired during the buildup of a (PLL_70-150KDa_-Fn)_10_film, after each adsorption of PLL_70-150KDa_ (blue) and after each adsorption of Fn (red). The amide I’ band (1600-1700 cm^-1^) and amide II’ band (1540-1600 cm^-1^) are represented. (B) Evolution of the Amide I’ band area measured by ATR-FTIR during multilayer films formation. The amide I’ band area measured after each PLL_70-150KDa_ adsorption is shown.

**
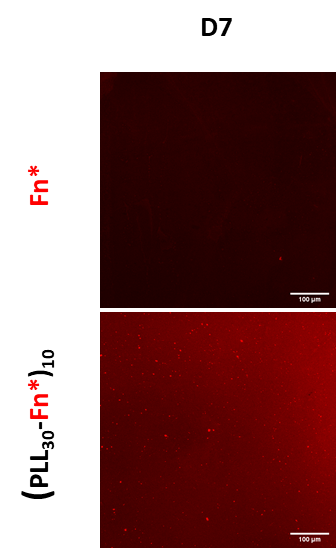
**

**Figure S2:** Fn* monolayer and (PLL_30_-Fn*)_10_ films after 7 days in cell culture media. Scale bar = 100 µm

**
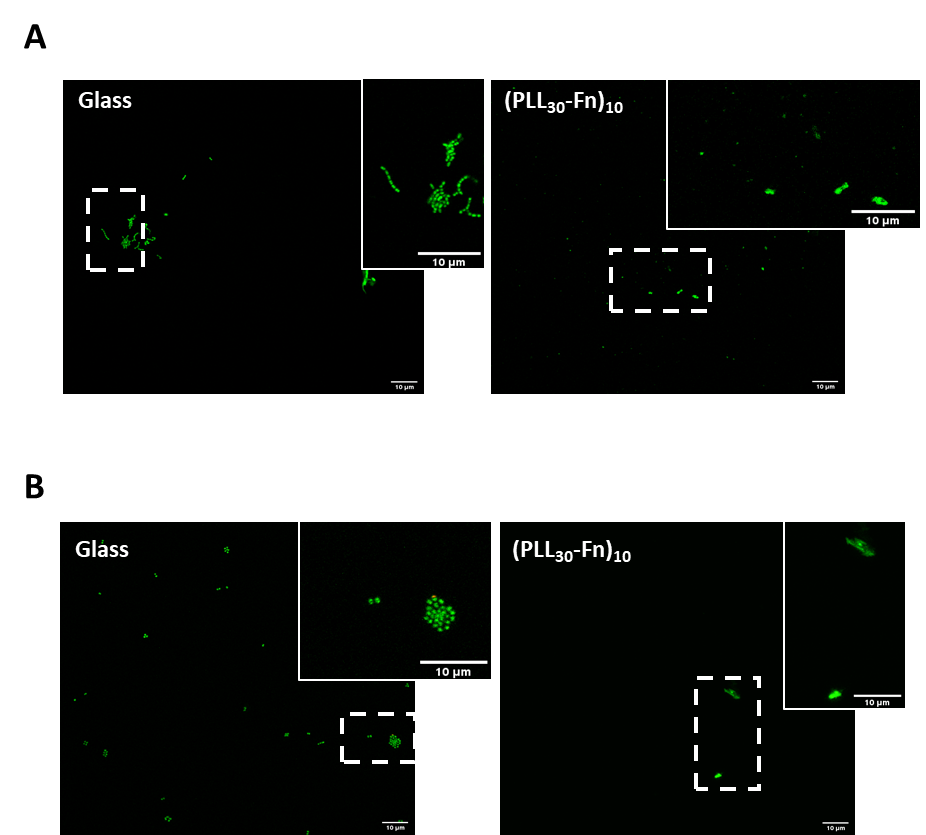
**

**Figure S3:** Representative images of LIVE/DEAD staining of adherent *S. mutans* (A) and *S. epidermidis* (B) on glass and (PLL_30_-Fn)_10_ films (n=3). The magnified captions correspond to the area surrounded by dashed rectangles in the original images. Note: Green indicates live bacteria, red indicates dead bacteria. Scale bar = 10 µm.
